# Optical coherence tomography: an introduction

**Published:** 2025-01-31

**Authors:** David Yorston

**Affiliations:** 1 Consultant Ophthalmologist, Tennent Institute of Ophthalmology, Gartnavel Hospital, Glasgow, Scotland.

**OCT scans support the diagnosis of macular conditions by allowing us to see the thickness of individual layers of the retina.**

In 1991, researchers at the Massachusetts Institute of Technology described a method using low intensity laser light to obtain an image of the retina. Because the system used light, it was called optical; because it used light from a laser source, in which the light is “coherent” – i.e. all the light waves are parallel and in step – they called it coherent. Because the image is a cross section, they called it tomography – ending up with the name optical coherence tomography or OCT.

OCT is convenient and comfortable for the patient: it only uses low-intensity light (it is much less bright than the light from an ophthalmoscope or slit lamp), it is not always necessary to dilate the pupil in order to obtain a satisfactory image, and the scanning process takes only a few seconds.

“OCT scans provide us with an extraordinarily detailed image of the central retina.”

The first OCT devices were available at the turn of the century, but they acquired images slowly and with limited detail, scanning the retina at a frequency of 400 sweeps per second. The ‘spectral domain’ OCT devices now in common use acquire images much more quickly, with a scanning frequency of over 25,000 sweeps per second, which gives a resolution of less than 10 microns. The very latest OCT machines are able to detect the movement of individual red blood cells in the retinal capillaries, which enables ophthalmologists to obtain very detailed images of the retinal circulation, called OCT-angiography (OCT-A). These devices can detect macular new vessels, new vessels of the retina, or the absence of capillaries in retinal ischaemia.

## How does OCT work?

The retina contains multiple layers of tissue of different densities. The OCT machine directs laser light at the retina, which is bounced off the different layers and the interfaces between them. A sensor gathers data about the reflected light by comparing the reflected light beam to a reference beam, which is reflected off a plain mirror. An image processing computer then converts the data into a usable image using a complicated algorithm which is beyond the scope of this article to explain.

OCT scans provide us with an extraordinarily detailed image of the central retina. We can measure the thickness of the macula and establish in which layer the abnormalities, such as fluid or exudate, are present. Changes in thickness can also be measured and monitored. Increased thickness may be due to age-related macular degeneration (AMD), diabetic maculopathy, or macular oedema (related to any other vascular or inflammatory diseases), and a reduction in thickness might be related to loss of tissue, such as geographic atrophy in AMD.

Some layers of the retina are **hyporeflective** – they don't reflect much light, so appear darker on OCT. The ganglion cell layer, which contains the cell bodies of the optic nerve cells, looks dark on OCT. Fluid, either within or under the retina, is also hyporeflective and therefore dark on OCT.

Other layers are **hyperreflective** – they reflect light, and so appear brighter on OCT. The ellipsoid zone – which represents the junction between the photoreceptor outer segments and the photoreceptor cell bodies – usually shows up as a bright white line. Exudates are also hyperreflective and appear white on OCT.

OCT is used most commonly to show a detailed cross-section of the central retina. In addition, it can image the optic nerve, and is therefore a useful tool to detect the loss of optic nerve fibres in glaucoma.

The ability of OCT to measure the thickness of the different retinal layers accurately makes it useful outside ophthalmology. For example, reductions in nerve fibre layer thickness are associated with neurodegenerative conditions such as Alzheimer's or Parkinson's disease. Early identification of people who may be at risk may allow preventive treatments to be given sooner, when they may be more effective.

**Figure 1:**
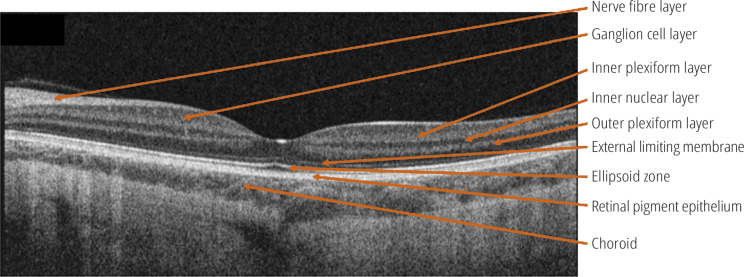
**Normal anatomy of the macula on OCT.** The photoreceptors are represented by the outer plexiform layer, external limiting membrane and the ellipsoid zone. The ellipsoid zone is the junction between the photoreceptor cell bodies and the outer segments of the photoreceptors. If it is absent, there are no photoreceptor outer segments present. The central depression is the foveal pit and is a normal feature of a healthy macula.

**Figure 2:**
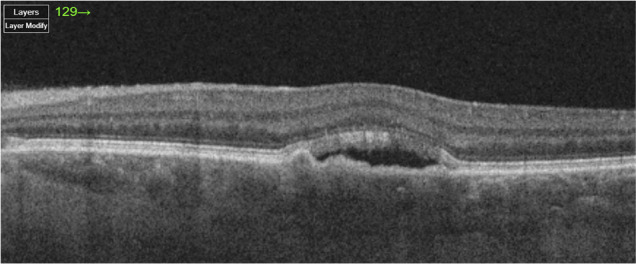
**Early exudative AMD.** There is sub-retinal fluid, and the retinal pigment epithelium is irregular. The fibrovascular scar tissue cannot be seen at this stage.

**Figure 3:**
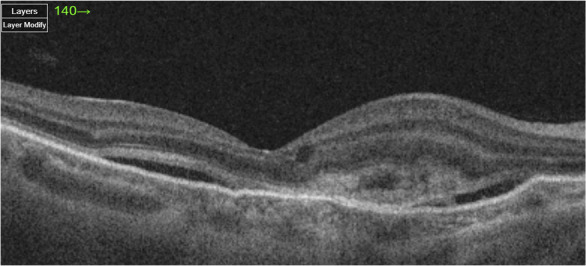
**Exudative AMD.** The OCT scan shows fluid under the retina. The sub-retinal neovascular membrane is seen to the right of the fovea.

**Figure 4:**
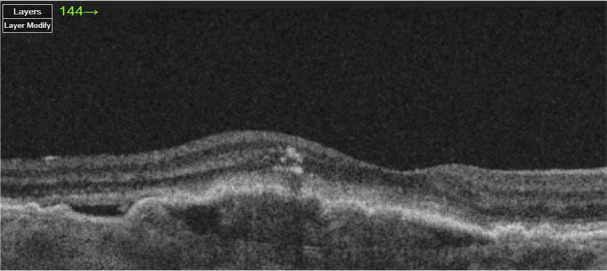
**Exudative AMD.** There is fluid under the retina, and the fibrovascular membrane can be seen under the retinal pigment epithelium. The hyperreflective white spots in the retina are exudates.

Although the physics and technology of OCT are complex, the basic skills for interpreting the images can be learnt relatively quickly. Figure 1 shows the normal anatomy of the macula; Figures 2–7 are examples of common macular conditions.

© DAVID YORSTON CC BY-NC-SA 4.0

© DAVID YORSTON CC BY-NC-SA 4.0

© DAVID YORSTON CC BY-NC-SA 4.0

© DAVID YORSTON CC BY-NC-SA 4.0

**Figure 5a:**
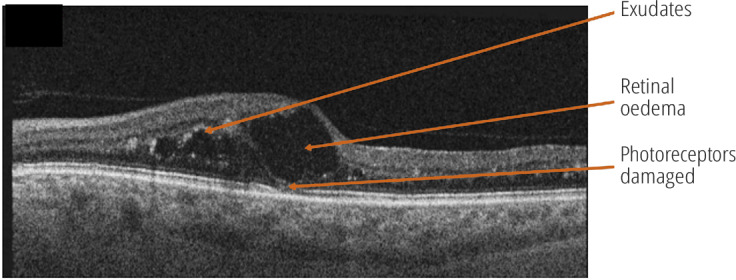
**Diabetic macular oedema**. The retina is thickened and oedematous. Unlike the thickening caused by the epiretinal membrane, all layers of the retina are affected. The larger white hyperreflective spots are hard exudates (the small ones are hyperreflective foci).

© DAVID YORSTON CC BY-NC-SA 4.0

**Figure 5b:**
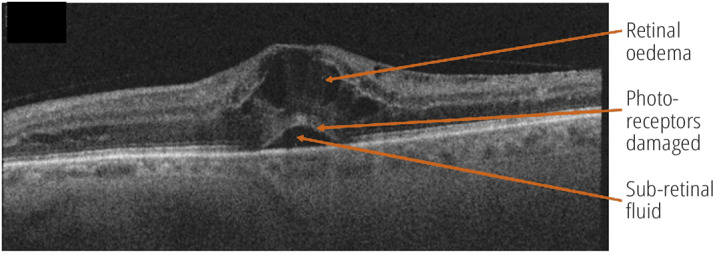
**Diabetic macular oedema.** The retina is thickened and elevated. Fluid has accumulated within the layers of the retina and under the fovea. The ellipsoid zone is not visible where the retina is most affected.

© DAVID YORSTON CC BY-NC-SA 4.0

**Figure 6:**
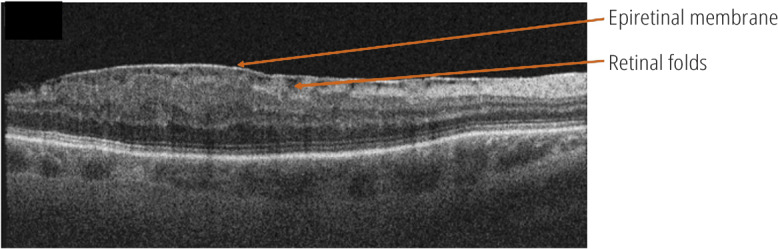
**An epiretinal membrane.** There is a hyperreflective membrane on the retinal surface. The inner retina is wrinkled and thickened, and the normal inner layers are not clearly defined. The photoreceptors and retinal pigment epithelium are unaffected.

© DAVID YORSTON CC BY-NC-SA 4.0

**Figure 7a:**
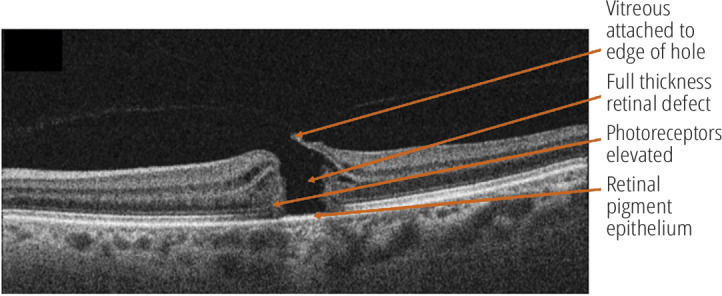
**A macular hole.** The scan shows complete loss of all retina layers, from the vitreous to the retinal pigment epithelium.

© DAVID YORSTON CC BY-NC-SA 4.0

**Figure 7b:**
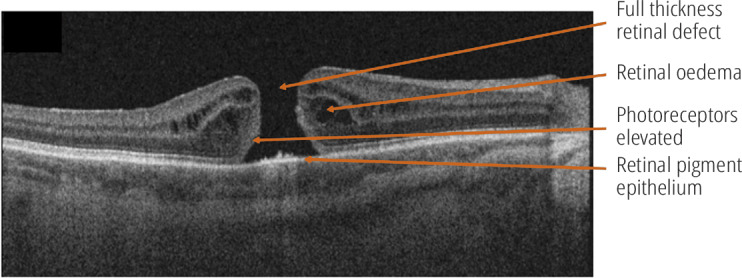
**A macular hole.** The vitreous is separated from the macular hole, and there are fluid-filled cysts within the retina, causing swelling of the retina surrounding the hole.

© DAVID YORSTON CC BY-NC-SA 4.0

